# Prison Discipline

**Published:** 1847-06

**Authors:** 


### PRISON DISCIPLINE.

We received a few days since a pamphlet relative to prison discipline, written by Mr. George Sumner, of Boston. Mr. Sumner has been several years in France engaged in the investigation of the subject, and has produced an able and unanswerable argument in favor of separate confinement. He has had access to ministerial documents abroad, and to every other source of information relative to the treatment of convicts, and what he urges must therefore have great weight with the authorities of his native city, to whom his letter is addressed.

The city council of Boston, in 1836, adopted a resolution declaring that it was incumbent upon the city government to provide a jail so constructed that each prisoner might be separately accommodated.

This principle, it seems, the council have abandoned, and the object of Mr. Sumner is to bring them back to what he conceives to be their duty. His array of facts is imposing, and, as we have said, his argument is one which, it appears to us, ought to carry conviction to the minds of the wise and good people of Boston. In one respect this is a medical question, and it is for this reason that the topic is introduced here: it has been urged against the system of separate confinement, that it is injurious to health, and predisposes especially to insanity. Mr. Sumner meets this objection by quoting the testimony of medical men and others in France who have been connected with the prisons in which this system has prevailed. We quote a part of it as follows: "From Bordeaux, at which place the separate prison of 168 cells has been occupied for four years, the prefect writes, 1st April, 1846: 'It is now fully established that the health of the prisoners is better under the separate system than under any other, that they receive with more fruit the consolations of religion, and that, not being excited by the bad example and counsels of their fellows, many reform, while none grow worse.' The physician writes that he was formerly disposed to consider this system as unfavorable to health, but his opinion has now changed. 'Only one original case of insanity,' he says, 'has occurred in the prison, and this was of a convict on receipt of the news of the rejection of his appeal for a new trial.' The visiting committee, composed of some of the most honorable and enlightened citizens of Bordeaux, write: 'For our own part, most of us having originally formed opinions unfavorable to the system of separation by day as well as by night, we deem it our duty to declare that *experience has proved we had fallen into error*; and that we consider the system of separate imprisonment, accompanied by labor, reading, religious services and daily walks—the system, in short, as it is practised at Bordeaux—as one of the reforms which reflect the greatest honor on our age.'"

Much more to the same point is adduced, which must effectually stop the mouths of antagonists on this point. So far from injuring, it is evident that the system has improved the health of prisoners in multitudes of instances. But we have not space nor time to pursue the subject, and must dismiss it with a single other extract:

"From Montpelier, the physician of the separate prison, who is also a professor of the distinguished medical faculty of that ancient town, writes: 'There are fewer maladies under the new system than under the old. *Pulmonary complaints have above all diminished.* The average number formerly was 31 cases in 121. Under the influence of the new system there have been only 17 in 112. Rheumatic affections have diminished one-half. The epidemics of different seasons do not penetrate the cells, while under the old system every disease in town was repeated in prison. The cases which occur are not only less numerous than before, but are of shorter duration. Prisoners who were feeble, emaciated and languishing on arrival, have acquired in a short time all the external signs of perfect health. Can any one doubt any longer of the good effects of the system on those who are well, when it aids so powerfully in restoring the health of those who are ill? Out of 658 men and 166 women received in the prison, 3 men and 1 woman have been put under treatment for mental derangement, but each one of them had shown signs of insanity before coming to the prison, and experience shows that the system of isolation, with its attendant visits, instead of increasing, has a tendency to moderate and quiet the predisposition to mental derangement.' "

Mr. Sumner's letter affords clear evidence of a vigorous mind, and, what is better, of a philanthropic heart. Pursuing the path in which Howard walked, may he live to accomplish like noble results.

